# Delivering acute stroke care in a middle-income country. The Mexican model: “ResISSSTE Cerebro”

**DOI:** 10.3389/fneur.2023.1103066

**Published:** 2023-02-22

**Authors:** Dulce María Bonifacio-Delgadillo, Enrique Castellanos-Pedroza, Bernardo Alfonso Martínez-Guerra, Claudia Marisol Sánchez-Martínez, Juan Manuel Marquez-Romero

**Affiliations:** ^1^Department of Interventional Neurology, Centro Médico Nacional 20 de Noviembre Instituto de Seguridad y Servicios Sociales de Los Trabajadores del Estado (ISSSTE), Mexico City, Mexico; ^2^Departament of Infectious Diseases, Instituto Nacional de Ciencias Médicas y Nutrición Salvador Zubirán, Mexico City, Mexico; ^3^Department of Neurology, Hospital General de Zona #2, Instituto Mexicano del Seguro Social (IMSS), Órganos de Operación Administrativa Desconcentrada (OOAD) Aguascalientes, Aguascalientes, Mexico

**Keywords:** acute ischemic stroke, stroke centers, hub-and-spoke, low- and middle-income countries, public health

## Abstract

**Introduction:**

Founded in 2019, the “ResISSSTE Cerebro” program is the first and only stroke network within the Mexican public health system. One advanced stroke center (ASC) and seven essential stroke centers (ESC) provide acute stroke (AS) care through a modified hub-and-spoke model. This study describes the workflow, metrics, and outcomes in AS obtained during the program's third year of operation.

**Materials and methods:**

Participants were adult beneficiaries of the ISSSTE health system in Mexico City with acute focal neurological deficit within 24 h of symptom onset. Initial evaluation could occur at any facility, but the stroke team at the ASC took all decisions regarding treatment and transfers of patients. Registered variables included demographics, stroke risk factors, AS treatment workflow time points, and clinical outcome measures.

**Results:**

We analyzed data from 236 patients, 104 (44.3%) men with a median age of 71 years. Sixty percent of the patients were initially evaluated at the ESC, and 122 (85.9%) were transferred to the ASC. The median transfer time was 123 min. The most common risk factor was hypertension (73.6%). Stroke subtypes were ischemic (86.0%) and hemorrhagic (14.0%). Median times for onset-to-door, door-to-imaging, door-to-needle, and door-to-groin were: 135.5, 37.0, 76.0, and 151.5 min, respectively. The rate of intravenous thrombolysis was 35%. Large vessel occlusion was present in 63 patients, from whom 44% received endovascular therapy; 71.4% achieved early clinical improvement (median NIHSS reduction of 11 points). Treatment-associated morbimortality was 3.4%.

**Conclusion:**

With the implementation of a modified hub-and-spoke model, this study shows that delivery of AS care in low- and middle-income countries is feasible and achieves good clinical outcomes.

## 1. Introduction

In Mexico, as in most low- and middle-income countries (LMICs), there is an enormous need for strategies to improve access to acute stroke (AS) care (1, 2). Intravenous thrombolysis (IVT) rate in Mexican hospitals is <10% (3), being the main reasons for its low utilization rate: patients arriving outside the therapeutic window (4), lack of knowledge regarding stroke symptoms and treatment in the Mexican population (5) and the documented fact that a significant proportion of patients undergo medical evaluations in facilities unable to provide adequate treatment before reaching an acute reperfusion capable center (3). Also, despite endovascular treatment (EVT) being the current standard of care for patients with acute large vessel occlusion (LVO), the access rate to EVT in Mexico is unknown, but a case series has shown it to be a feasible intervention with efficacy comparable to centers in high-income countries (6). Nevertheless, the widespread use of EVT in the country faces several barriers that still need to be overcome, mainly the high costs and the lack of public funding (7).

Regrettably, the number of new stroke cases and deaths in Mexico increased by 70.7 and 75.3%, respectively, from 1990 to 2019. And although the age-standardized mortality and the disability-adjusted life years rates were reduced −41.6 and −38.1%, respectively, in the same period, the burden of stroke continues to be a significant healthcare issue for the country (8).

To address this high burden of disease, in 2019, the “*Instituto de Seguridad y Servicios Sociales de Los Trabajadores del Estado*” (ISSSTE), which provides healthcare for the employees of the Mexican government and to their first-degree relatives, approved the creation of a pilot program; “ResISSSTE Cerebro,” a publicly funded program that provides AS care for the beneficiaries of the ISSSTE healthcare system in Mexico City. Before the program's implementation, data from the epidemiology department of the ISSSTE puts AS treatment rate at <10% (Unpublished data).

In this study, we report the results from the third year of operation of the “ResISSSTE Cerebro” program, including the population's characteristics, treatment, performance metrics, and early clinical outcomes.

## 2. Materials and methods

### 2.1. The “ResISSSTE Cerebro” program

The “ResISSSTE Cerebro” program includes seven urban healthcare facilities located in Mexico City and one in each of the neighboring states of Morelos and Hidalgo. According to the World Stroke Organization global stroke services guidelines and action plan (9), seven facilities are cataloged as essential stroke centers (ESC). Thus they offer access to non-contrast computed tomography (NCCT), clinical evaluation, and potentially IVT (according to IVT criteria cited below). At ESC, there is no personnel with expertise in AS treatment. The eighth facility is an advanced stroke center (ASC) capable of providing advanced stroke services on a 24/7 basis, including multidisciplinary stroke expertise, multimodal imaging, and acute reperfusion therapies for ischemic stroke.

Since its approval in 2019, the program has operated as a modified hub-and-spoke model. It receives funding from the Mexican government through the ISSSTE healthcare system and has access to ambulance services available 24/7. It also includes a stroke telemedicine network to facilitate the evaluation and care of potential patients.

As mentioned above, the program's functioning is mainly based on the hub-and-spoke model but with certain adequations to the Mexican Healthcare system. For example, most hub-and-spoke models function by offering daytime AS treatment at local centers, and the patients in need of treatment out-of-hours and on weekends are treated at hub hospitals. But, in the “ResISSSTE Cerebro” program, all centers provide AS treatment regardless of time or day, with the only difference being that advanced modalities of treatment (EVT and IVT guided by perfusion imaging up to 9 h after the onset of symptoms) are available only at the ASC. Similarly, the drip-and-ship model, as initially conceived, assumes that all centers within a network can diagnose LVO, thus allowing emergency medical services (EMS) to move patients to the closest hospital and only transfer to a thrombectomy-ready hospital for those patients with confirmed LVO. The drip-and-ship model was only partially implemented in our program due to constrained access to ambulances and human and technological infrastructure to perform advanced imaging in stroke patients at the ESC. Our model also accommodates that most of the patients in Mexico arrive at a hospital by their means (for example, the family car or public transportation), with few coming by EMS; therefore, prenotification is uncommon. Consequently, by concentrating the human and technological resources in a single center, the “ResISSSTE Cerebro” program can deliver advanced AS treatment 24/7 while preserving the capability of ESC to provide telemedicine supervised IVT also 24/7.

**Figure 1:**
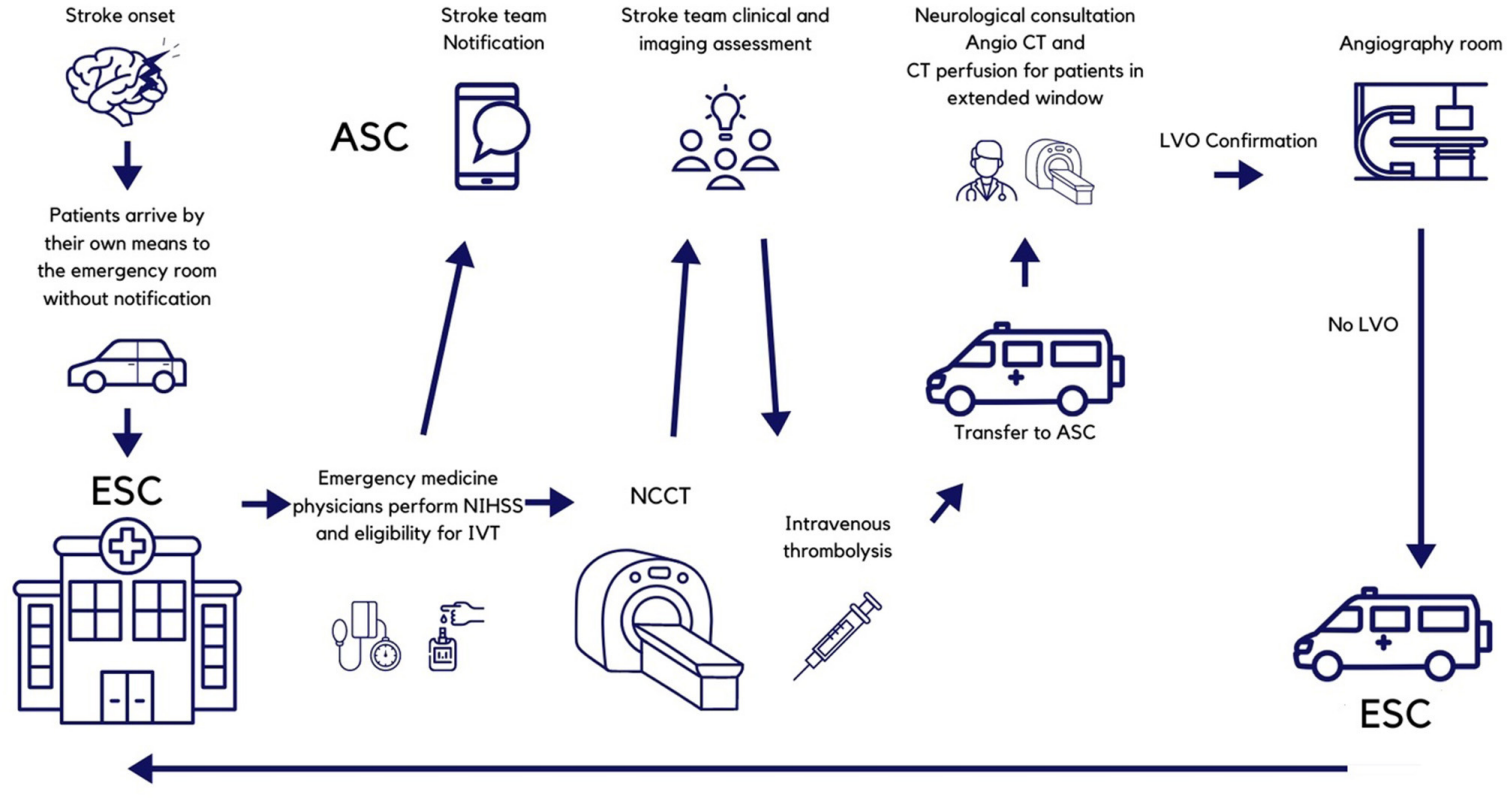
Pathway for patients initially arriving to Essential Stroke Centers. NIHSS, National Institutes of Health Stroke Scale; ASC, Advanced Stroke Center; IVT, intravenous thrombolysis; CT, computed tomography; LVO, large vessel occlusion.

**Figure 2:**
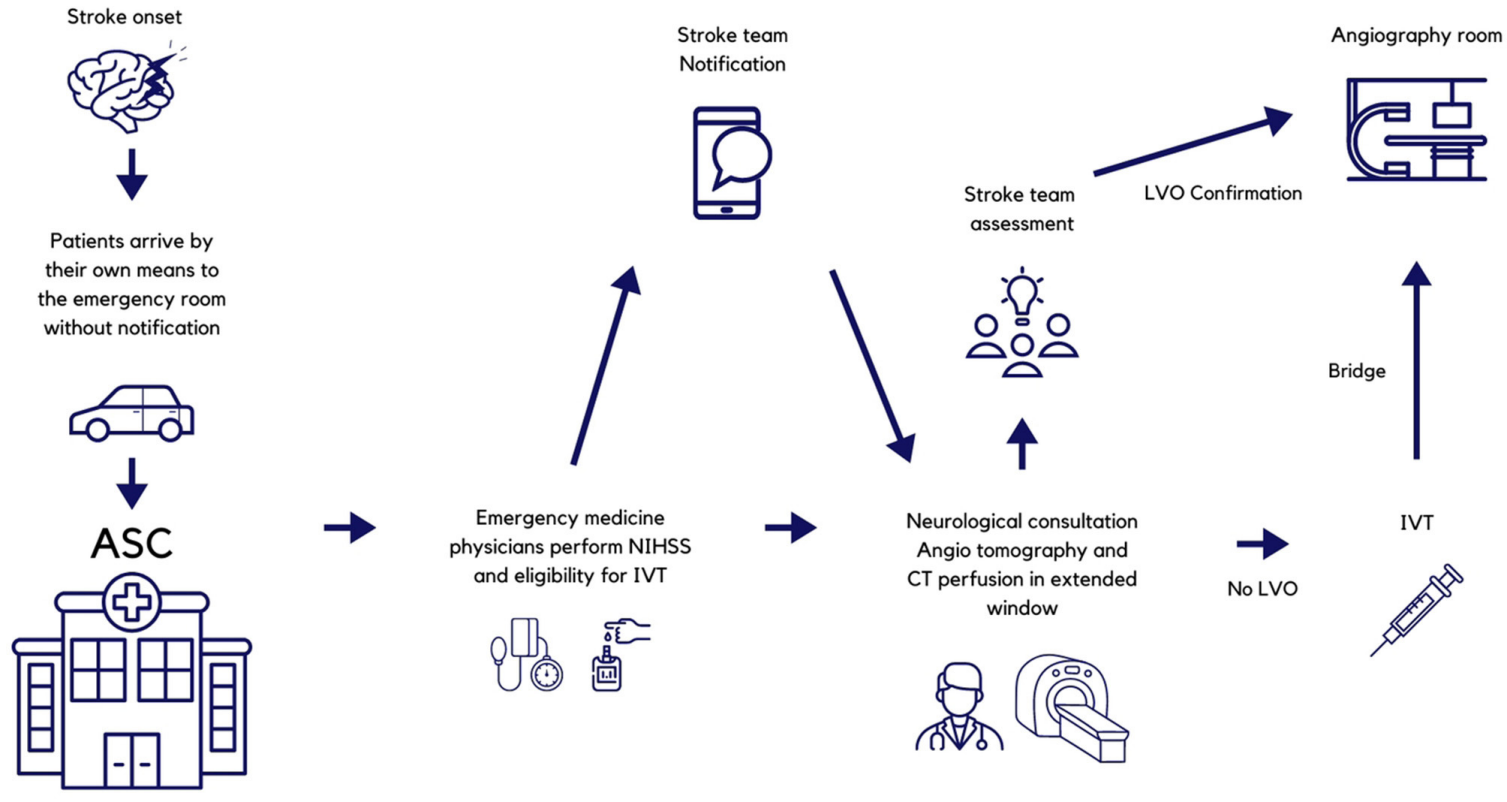
Pathway for patients arriving directly to the Advanced Stroke Center. NIHSS, National Institutes of Health Stroke Scale; ESC, Essential Stroke Center; ASC, Advanced Stroke Center; IVT, intravenous thrombolysis; CT, computed tomography; LVO, large vessel occlusion.

The stroke telemedicine network utilizes an instant messaging app that includes all the emergency room staff of all shifts grouped by each ESC. Each group, in turn, has all the stroke team members located at the ASC. Emergency room physicians are in charge of all initial evaluations and are responsible for alerting the stroke team and carrying out their instructions regarding treatment. At the same time, they order the NCCT and arrange for a possible transfer to the ASC. The ESC prenotifies all transfers to ASC. The protocol is known by all the staff at the emergency rooms of the ESC, and a print or electronic copy is available for consultation at the office of the head of the emergency department. Figure 1 depicts the pathway for patients initially arriving at ESC, and Figure 2 is that of patients coming directly to the ASC. All the ESCs are staffed 24/7 with emergency physicians, residents (emergency medicine is a 3-year residency program in Mexico), and radiologists. At the ASC, the staff comprises emergency physicians and emergency medicine residents, radiologists, neuroradiologists, clinical neurologists and clinical neurology residents, neurosurgeons and neurosurgery residents, and interventional neurologists and interventional neurology residents.

#### 2.1.1. Improvement and maintenance of the program

The program has implemented continuing medical education activities at the ESC. These activities include lectures and seminars for emergency department personnel, clinical rotations at the ASC for emergency medicine, written protocols for managing data, patient transfer, neuroimaging protocols, and flow charts. At the ASC, improvement measures included written protocols for collecting data, neuroimaging protocols, and flow diagrams for the separate treatment windows of treatment for acute ischemic stroke up to 24 h from symptom onset.

The program also takes notice of the increasing importance of routine monitoring of the quality of stroke care (10). Consequently, the ASC participates in the RES-Q initiative of the ESO East Project (European Stroke Organization-Enhancing and Accelerating Stroke Treatment) and registers all the patients treated, and conducts annual reviews of the gathered data (11). Additionally, in March 2021, an interventional neurology residency program was initiated. Its first class is expected to graduate in February 2023. One example of the continuing medical education implemented is the change in the use of tenecteplase. Since the recruitment period for the preset study predates the publication of the Norwegian tenecteplase stroke trial results, the dose for IV tenecteplase was set at 0.4mg/kg according to local practices.

Nevertheless, since the NORTEST trial publication (12) the “ResISSSTE Cerebro” program protocol has been modified, and the current dose is set at 0.25 mg/kg. Additionally, although tenecteplase is available at all the network centers, the protocol establishes that Alteplase is always the drug of choice, with tenecteplase reserved for rare occasions when Alteplase is unavailable. The low usage of tenecteplase in the present study supports the practice. Additionally, it is worth mentioning that as a result of all the strategies for improvement and maintenance, the main center was certified by the World Stroke Organization as an ASC in August 2022.

### 2.2. Selection of the acute reperfusion intervention

Patients 18 years or older arriving within the 4.5 h time window are eligible for IVT at the ESC after the evaluation of the NCCT by the stroke team at the ASC *via* telemedicine. Patients evaluated outside the 4.5-h time window are transferred to the ASC, undergoing brain computed tomography perfusion imaging combined with head and neck computed tomography angiography. Based on the results of advanced neuroimaging, extended window IVT (up to 9.0 h from symptoms onset) is offered according to the EXTEND—IA criteria using RAPID—AI software or EVT (up to 24 h) using DEFUSE −3 or DAWN criteria. Wake-up stroke and stroke of unknown onset are offered treatment based on the EXTEND and ECASS4—EXTEND trial for up to 9.0 h IVT is administered at standard doses, 0.9 mg/kg for Alteplase, a single bolus of 0.4 mg/kg for Tenecteplase, and 0.25 mg/kg single bolus of Tenecteplase when subsequent EVT was planned.

The EVT technique was at the discretion of the attending neuro-interventionalist and consisted of direct aspiration, stent retrievers, or a combination.

### 2.3. Data and outcomes

Data was prospectively registered from March 1st, 2021, to February 28th, 2022. Stroke severity was determined by the National Institutes of Health Stroke Severity scale (NIHSS) with minor symptoms defined as NIHSS 0 −5 and severe stroke if NIHSS > 25 points. Successful recanalization was defined as a modified Thrombolysis in Cerebral Infarction (mTICI) score of 2b−3. Early neurological improvement (ENI) was defined as a reduction of ≥4 on the National Institutes of Health Stroke Scale (NIHSS), compared with the baseline score or an NIHSS of 0 or 1 at 24 h after treatment; European Cooperative Acute Stroke Study (ECASS) II criteria were used to define any intracranial hemorrhage and symptomatic intracranial hemorrhage (sICH) after t-PA administration. The compound measure of adding mortality and morbidity is reported as morbimortality.

#### 2.3.1. Workflow times definitions

We utilized standardized definitions for workflow times except for the door-to-evaluation time, which was not included because, for most of the patients evaluated initially at the ESC, the time to the medical evaluation was registered irregularly. Instead, we used door-to-imaging (DTI) time (measured from arrival at the hospital to the arrival at the imaging suite) as an estimate of the time that passed until the assessment of the patient since the initial evaluation forcibly had to be complete before the transfer to radiology. Onset-to-door (OTD) was measured from the onset of symptoms to the arrival at the hospital regardless of if it was an ESC or the ASC. The time from arrival at the hospital to the starting of IVT is reported as door-to-needle (DTN), whereas the time from the onset of symptoms and arrival at the ASC to groin puncture is reported as onset-to-groin (OTG) and door-to-groin (DTG), respectively. Finally, the transfer time (TT) was measured from the stroke team notification to the patient's arrival at the ASC.

### 2.4. Statistical analysis

Categorical variables are presented as frequency and percentages, continuous variables as mean ± standard deviation or median (second–third quartiles) according to the Kolmogorov–Smirnov test result.

As appropriate, comparisons were conducted using a chi-squared test, Fisher's exact test, Student *T*-test, and Mann–Whitney-*U*-test. The *p*-value was considered significant at <0.05. All analyses were computed with Stata version 15.0, StataCorp (Texas, USA).

### 2.5. Ethical considerations

The institutional review board of the ISSSTE, Centro Médico Nacional “20 de Noviembre,” reviewed and approved the protocol (reference 582.2019). The same committee waived the signing of the informed consent form per local regulations. Only de-identified data were registered and stored.

## 3. Results

### 3.1. Baseline characteristics of the patients

**Figure 3:**
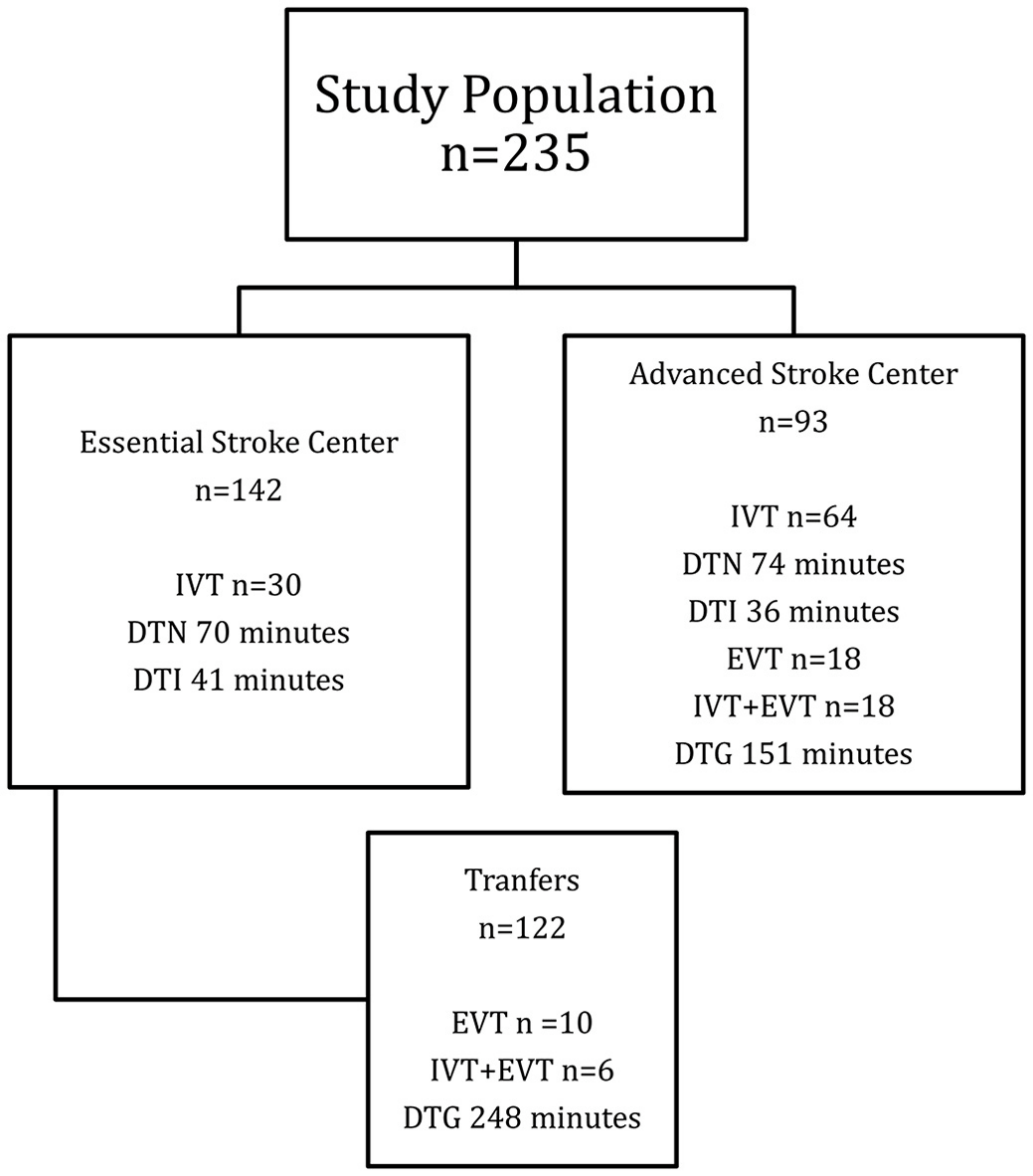
Flow chart of study population. IVT, intravenous thrombolysis; DTN, door-to-needle; DTI, door-to-imaging; EVT, endovascular therapy; DTG, door-to-groin.

Data from 235 patients were available for the analysis; 104 (44.3) were men with a median age of 71 (60–78) years. One hundred and forty-two (60.4%) patients received their initial evaluation at one of the seven ESC of the stroke network, and 122 (85.9%) of them were transferred to the ASC for further assessment with a median transfer time (TT) of 123 min. Figure 3 depicts the flow chart of the study population. Ninety-three (39.6%) patients arrived directly at the ASC for urgent care. Vascular risk factors identified in order of frequency were hypertension 173 (73.6%), diabetes mellitus 81 (34.5%), obesity 40 (17.0%), cancer 20 (8.5%), valvular heart disease 14 (6.0%) and smoking 13 (5.5%). After clinical and imaging evaluation, 63 (26.8%) patients were identified with a stroke mimic. Subtypes of stroke were as follows: ischemic stroke 139 (80.8%), hemorrhagic stroke including intracerebral hemorrhage and subarachnoid hemorrhage 24 (14.0%), and nine transient ischemic attacks (5.2%). The median baseline NIHSS was 10 (4–18). Eighty-seven (61.3%) patients and 41 (44.1%) patients arrived within the 4.5 h therapeutic window to the ESC and ASC, respectively. As shown in Table 1, for baseline characteristics, overall, there were no differences between patients whose care took place in an ESC compared to the ASC except for previous disability (*p* = 0.044), frequency of any cancer, and valvular heart disease (*p* <0.001), baseline NIHSS (*p* = 0.028) and therapeutic window arrival (*p* = 0.044).

**Table 1:** Baseline characteristics of the patients.

	**All *n* = 235**	**ESC *n* = 142**	**ASC *n* = 93**	***p*-value**
Male	104 (44.3)	67 (47.2)	37 (39.8)	0.264
Age—median (2nd−3rd quartiles)	71 (60–78)	70.5 (60–79)	72 (62–78)	0.273
Previous stroke	30 (12.9)	20 (14.1)	10 (11.0)	0.479
**Modified Rankin Scale**				
0	168 (72.1)	111 (78.7)	57 (62.0)	0.042
1	27 (11.6)	12 (8.5)	15 (16.3)	
2	9 (3.9)	4 (2.8)	5 (5.4)	
3	16 (6.9)	9 (6.4)	7 (7.6)	
**Risks factors**				
Hypertension	173 (73.6)	104 (73.2)	69 (74.2)	0.871
Diabetes	81 (34.6)	56 (39.4)	25 (27.2)	0.054
Obesity	40 (17.1)	26 (18.3)	14 (14.2)	0.539
Cancer	20 (8.6)	2 (1.4)	18 (19.6)	<0.001
Valvular heart disease	14 (6.0)	2 (1.4)	12 (13.0)	<0.001
Smoking	13 (5.6)	10 (7.0)	3 (3.3)	0.257
**Type of stroke**				
Ischemic	139 (59.1)	93 (65.5)	46 (49.5)	0.096
Hemorrhagic	24 (10.2)	14 (9.9)	10 (10.8)	
Transient ischemic attack	9 (3.8)	4 (2.8)	5 (5.4)	
Large vessel occlusion	63 (26.8)	63 (26.8)	0	–
NIHSS—median (2nd−3rd quartiles)	10 (4–18)	11 (4–18)	8 (1–17)	0.028
**Time of arrival**				
<4.5 h	128 (54.5)	87 (61.3)	41 (44.1)	0.044
4.5–6.0 h	18 (7.7)	10 (7.0)	8 (8.6)	
6–16 h	22 (9.4)	9 (6.3)	13 (14.0)	
16–24 h	2 (0.9)	1 (0.7)	1 (1.1)	
>24 h	1 (0.4)	0	1 (1.1)	

All values n (%) unless otherwise specified.

NIHSS, National Institutes of Health Stroke Scale; ESC, Essential Stroke Center; ASC, Advanced Stroke Center.

### 3.2. Time points for acute stroke treatment

Performance time points achieved were as follows:

OTD median time 135.5 (90–270) min.
DTI median time 37 (26–52) min.
DTN median time 76 (40–133) min, and
DTG median time of 151.5 (118–225) min.

Table 2 shows comparisons in the time points for acute stroke treatment workflow between ASC and ESC patients. Forty-nine (35.3%) patients received IVT at either center. Thrombolysis was achieved within the first hour of arrival to the emergency department in 15 (30.6%) patients (Table 3).

**Table 2:** Time points for acute stroke treatment workflow.

	**ESC**	**ASC**	***p*-value**	**Transferred**	***p*-value**
OTD time	127.5 (90–195)	182.5 (93–354)	0.0813		
DTI time	41 (23–81)	58 (26–52)	0.0582	30.5 (40–92)	<0.001
DTN time	70 (40–134)	97 (45–97)	0.5067	67.5 (47–76)	0.1568

All values median (2nd−3rd quartiles). All values in minutes unless otherwise specified.

ESC, Essential Stroke Center; ASC, Advanced Stroke Center; OTD, onset-to-door; DTI, door-to-imaging; DTN, door-to-needle.

**Table 3:** Comparison of overall and within the first hour intravenous thrombolysis rates by treatment center.

	**All**	**ESC**	**ASC**	***p*-value**
IVT	49 (35.3)	30 (61.2)	19 (38.8)	0.558
1st hour IVT	15 (30.6)	9 (30.0)	6 (31.6)	0.452

All values n (%).

ESC, Essential Stroke Center; ASC, Comprehensive Stroke Center; IVT, intravenous thrombolysis.

### 3.3. Efficacy and safety of intravenous thrombolysis

After IVT, 35 patients (71.4%) showed ENI with a median NIHSS reduction of 11 points (4–16). Hemorrhage after intravenous thrombolysis presented in eight patients, but only two were symptomatic. Unfortunately, both cases produced the patient's death, and one additional death occurred with a mortality rate of 6.1%. All the patients that developed hemorrhage received Alteplase. Three patients received tenecteplase (6.1%).

### 3.4. Endovascular treatment

The ASC performed 28 endovascular procedures. The median OTG time was 500 min (353–792), and the median DTG time was 151.5 min (118–225). Seven (25%) patients were treated within the 6-h window. One patient arrived at the ASC beyond the 24-h windows but was still eligible for EVT. According to the study design, all other patients received treatment in the 6-to-24 h therapeutic window per protocol.

## 4. Discussion

The present study constitutes the first description of an AS treatment program in Mexico; its results show adequate performance in standardized workflow metrics in AS treatment and good immediate clinical outcomes.

When there is a need to decide whether to treat *in situ* or transfer a patient AIS, two main issues are essential: (1) prolonged transfer times will result in delays in IVT administration which are associated with significant morbidity and mortality, and (2) patients carrying LVO (~10%−46% of the cases), will significantly benefit from EVT (1, 13). Therefore, it is critical to consider the regional infrastructure available along with the location of ESC and ASC concerning the patient's geographical location (14). In this respect, Bekelis et al. consider bypassing primary stroke centers making sense if the center is located within 90 min from the patient's location. But they also did not find any differences between patients treated with a hub-and-spoke model and those treated directly in ASC in several outcomes, including inpatient case-fatality, discharge to a specialized facility, and length of stay (13).

In other studies, the hub-and-spoke model has been associated with better outcomes, highlighting that rapid inter-facility transfer is a pivotal key to the efficacy of such a model (15). Milne et al. (14) demonstrated that ESC near an ASC retained their significance through the drip and ship approach if they could administer IVT within 30 min. Prior experiences in LMIC of successful development and implementation of stroke units have been reported in the private setting of Panamá, but it was a single-center program (16). Considering all these factors, our local infrastructure, capabilities, and the geographical distribution of the healthcare facilities, we decided to adopt the hub-and-spoke model. The results for the third year since the implementation of the program show higher transfer times compared to similar programs [123 vs. 104 min in the study by Prabhakaran et al. (15)].

According to national epidemiological data, the ISSSTE healthcare system covers only a tiny proportion of the Mexican population (11.3%) (17). Regardless, in our population of beneficiaries, we observed similar demographics, risk factors, and proportion of stroke subtypes than reported in previous studies performed in Mexico (18). Regarding AS treatment rates before the introduction of the extended time window, IVT and EVT range from 0.5 to 7.6% (4, 18–22). In light of these figures, the results of the present study show a substantial improvement in rates of IVT and EVT (35.3%), something unprecedented for the country. Moreover, the “ResISSSTE Cerebro” program differs from previous efforts in Mexico due to its multicenter nature, usage of telemedicine, and public origin of the funding source; these differences allow for bypassing common barriers to treatment of AIS, such as low availability of stroke expertise, thrombolytics, angiography suites, and endovascular devices, and uncoordinated transfer protocols. Lastly, its most crucial facilitator is the availability of public funding to support the interventions.

We also significantly improved in time metrics. OTD diminished from 11 h to 135.5 min. Our program's arrival times were lower, with 54.5 and 62.1% arriving within the 4.5- and 6.0-h windows, respectively. Previous studies had reported 17%−23% of patients coming in <3.0 h (21, 22), 17.4% coming in <4.5 h (17), and 39%−42% within 6 h from symptoms onset (19, 22). Remarkably, there were no differences in patients' arrival time between ESC and ASC. Also, the ESC administered most of the IVT; this situation is optimal because it allows for complex cases to be treated in the ASC (23).

Besides OTD, the present study's most important finding is that there were no differences in DTN times between the ESC and the ASC, demonstrating that the program can provide timely care decisions regardless of the physical infrastructure or geographical location of the stroke team. Still, we detected a non-significant (~30 min) delay in IVT for patients arriving at the ASC without prenotification.

The good performance in time measures after IVT also translated into excellent early efficacy outcomes, with 71.4% of the patients achieving ENI, as evidenced by their post-IVT NIHSS scores. Concerning safety, the rate of symptomatic intracerebral hemorrhage and in-hospital deaths was lower than those previously reported for public healthcare facilities in Mexico (19). Interestingly, transferred patients from ESC had a shorter DTN when compared to patients that arrived directly at the ASC. Similarly, DTG in the RACECAT trial (24) was higher in the thrombectomy-capable center than in the local stroke center, 71 (49–97) vs. 43 (32–59) min, respectively. The time differences, although not statistically significant, underline the importance of specific processes, such as the prenotification system and the stroke team's activation, and give us the cue to develop further and implement strategies to improve the program. Some methods we could implement to reduce performance time measures include critical components of the Helsinki model, such as those successfully implemented in other parts of the world (25). For example, the direct transfer of patients from triage onto the CT table on the ambulance stretcher; and the delivery in the CT room immediately after imaging. Others, such as ambulance prenotifications, will continue to be a challenge in Mexico due to the general population's low usage of EMS.

Despite all the positive findings, the present study has some limitations, mainly the data that was not registered; these data included door-to-evaluation time, transfer selection criteria, mechanism of stroke, time to start secondary prevention strategies, and long-term clinical outcomes. We have also previously identified long transfer times as a significant cause for delays in DTG and DTI times at the ASC (26). The prolonged transfer times are explained majorly by two factors: the use of ambulances at ESC for pre-scheduled transfers without a backup ambulance for emergency transfers and the fact that Mexico City is the world's most traffic-congested city. To tackle the first issue, we strongly required the ISSSTE board to designate at least one ambulance per ESC exclusive to the “ResISSSTE Cerebro” program; however, funding and logistic matters still prevent such improvement to our program.

Additionally, the generalizability of our findings is restricted to the beneficiaries of the ISSSTE living in the urban setting of Mexico City. In other cities of the country or different LMICs, the scarcity of resources, lack of organized communication among facilities, and restricted access to technology make the widespread implementation of our program non-viable at the present moment.

Regardless, the present study serves as an initial experience that provides evidence that implementing programs aimed to reduce the burden of stroke in LMICs is possible and has a high chance of success, provided that adequate funding is available. In the design of the “ResISSSTE Cerebro” program, we incorporated multiple strategies proven to increase rates of AIS treatment (27), such as education programs, enabling access to stroke expertise and technology, and timely communication in a stroke network.

## 5. Conclusion

The “ResISSSTE Cerebro” program is a successful AS treatment model capable of achieving high rates of IVT and EVT for the treatment of AIS within its third year of operation. Also, workflow metrics were within international standards and, for IVT, did not differ between ESC and ASC. The previous results translated into ENI for most patients without increases in morbimortality. We are confident that in LMICs, implementing programs like “ResISSSTE Cerebro” can lead to the successful delivery of AS care.

## Data availability statement

The raw data supporting the conclusions of this article will be made available by the authors, without undue reservation.

## Ethics statement

The studies involving human participants were reviewed and approved by Institutional Review Board of the ISSSTE, Centro Médico Nacional XX de Noviembre. Written informed consent for participation was not required for this study in accordance with the national legislation and the institutional requirements.

## Author contributions

DB-D design and conceptualization of the study, significant role in acquiring data, analyzed the data, and drafted the manuscript for intellectual content. EC-P analyzed the data and drafted the manuscript for intellectual content. BM-G and CS-M had a substantial role in the acquisition of data, analyzed the data, and drafted the manuscript for intellectual content. JM-R the study's conceptualization, analyzed the data, and drafted the manuscript for intellectual content. All authors contributed to the article and approved the submitted version.
